# Analysis of comb morphology in Sichuan Mountaineous Black-bone chickens and its correlation with growth performance

**DOI:** 10.1016/j.psj.2025.105168

**Published:** 2025-04-17

**Authors:** Kunlong Qi, Juan He, Felix Kwame Amevor, Zheliang Liu, Chencan Zhai, Yingjie Wang, Liuting Wu, Gang Shu, Xiaoling Zhao

**Affiliations:** a State Key Laboratory of Swine and Poultry Breeding Industry, College of Animal Science and Technology, Sichuan Agricultural University, Chengdu, Sichuan, PR China; b Farm Animal Genetic Resources Exploration and Innovation Key Laboratory of Sichuan Province, College of Animal Science and Technology, Sichuan Agricultural University, Chengdu, Sichuan, PR China; c Key Laboratory of Livestock and Poultry Multi-omics, Ministry of Agriculture and Rural Affairs, Sichuan Agricultural University, Chengdu, Sichuan, PR China; d Department of Basic Veterinary Medicine, Sichuan Agricultural University, Chengdu, Sichuan, PR China

**Keywords:** Sichuan Mountaineous Black-bone chicken, Comb development, Growth performance, Histomorphology, Growth hormone

## Abstract

Biological ornaments, such as the comb in chickens, act as condition-dependent indicators of mate quality and are influenced by genetic, hormonal, and environmental factors. In this study, we investigated the histomorphological development of combs in Sichuan Mountaineous Black-bone chickens and examined their relationship with growth performance and hormone levels. At market age, the chickens were classified into large- and small-comb groups based on comb size, and their skeletal traits, slaughter performance, and meat quality were evaluated. The results showed that male chickens with large combs exhibited significantly better skeletal development, including greater shank circumference, breast depth, and breast circumference, although slaughter traits relative to body weight did not differ significantly. In female chickens, a similar pattern was observed in the large-comb group, but the differences were not statistically significant. Meat quality analysis revealed that male chickens with large combs had higher pH and moisture in breast muscle, while crude protein and crude fat were higher in those with smalle combs. L* value and inosine monophosphate (IMP) were more abundant in the breast muscle of female chickens with large combs. Growth hormone (GH) levels were positively associated with comb traits in both sexes, whereas testosterone showed no significant correlation. Gene expression analysis indicated that *BMP2* and *HSD17B2* were upregulated in small-comb chickens, while chondroadherin-like (*CHADL*) was upregulated in large-comb chickens. These findings enhance our understanding of the biological basis of comb development and its link to growth performance, offering useful insights for improving productivity in poultry through ornamental trait selection and economic and scientific values.

## Introduction

Biological ornaments are distinctive traits that often function as condition-dependent indicators of mate quality during selection (McCullough et al., 2016; Nolazco et al., 2022). In poultry, the comb is a prominent ornamental feature and a key factor influencing consumer preferences for live birds. As a secondary sexual characteristic in chickens, comb development has been linked to sexual maturity and reproductive performance (Wright et al., 2012). The comb size and morphology are regulated by a complex interplay of genetic, hormonal, and environmental factors, including breed, photoperiod, gene expression, and endocrine activity (Osol et al., 1980; Fölsch et al., 1994; Bakovic et al., 2022). The comb also serves as a practical indicator of health status and reproductive performance. Abnormally pale combs may signal anemia or parasitic infections, while swollen or necrotic combs may indicate infections or frostbite (Hoerr et al., 1982). In male chickens, comb size is positively associated with testicular size and sperm quality. Beyond its physiological roles, chicken combs have potential biomedical applications (Navara et al., 2012; Rizzi and Verdiglione, 2015; Łukaszewicz et al., 2018). Comb-derived extracts have been shown to improve joint function in athletes and serve as a rich source of hyaluronic acid, which is used in ophthalmic surgery (Yoshimura et al., 2012; Higashide and Sugiyama, 2008). Therefore, the comb is a multifunctional structure with physiological, behavioral, and health-related functions, in chickens, alongside applications in human health.

Comb size is highly heritable, reflecting strong genetic control (Shen et al., 2016). A study identified that 280, 201, and 278 QTLs were related to comb length, height, and weight, respectively, as of December 27, 2024 (Hu et al., 2016). Genes such as *HAO1* and *BMP2* have been implicated in comb development (Liu et al., 2018). Despite these advances, there remain gaps in our understanding of comb development in relation to hormone levels, sex-specific growth patterns, and their links to production traits (Eitan et al., 1998). The developmental patterns and morphological characteristics of the comb in relation to growth performance have been minimally studied. Hence, the present study investigates the histomorphological development of the chicken comb and explored its associations with growth performance, meat quality, and hormone levels. By elucidating the biological mechanisms underlying chicken comb development, the findings from this study will enhance our understanding of its functional significance and potential role in improving poultry productivity.

## Materials and methods

### Animals and tissues

The Sichuan Mountaineous Black-bone chickens used in this study were provided by Sichuan Fengyan Muye Agricultural Development Co., Ltd., with its central production area located in Xuyong County, Luzhou City, Sichuan Province. This breed has a long history of domestication, dating back more than 300 years to the Kangxi dynasty period. Sichuan Mountaineous Black-bone chicken is a local chicken breed, it is characterized by black skin, black bones, shiny black feathers, and strong adaptability. Both its meat and bones can be used in medicine, offering benefits such as nourishing yin, dispelling dampness, regulating menstruation, and replenishing blood.

The chickens used in this study were all single comb type, 12 chickens were randomly selected and euthanized for sample collection. Comb tissues were collected and immediately transferred to liquid nitrogen, then stored at −80°C for subsequent RNA extraction. The animal experiments were approved by the Institutional Animal Care and Use Committee of Sichuan Agricultural University (SYXK2019-187). All experiments were conducted in accordance with the guidelines provided by the Animal Welfare and Ethics Committee of Sichuan Agricultural University.

### Measurement of comb and body weight development

At 0 d, 28 d, 42 d, 98 d, 138 d, 180 d, 240 d, and 476 d, 6 individuals with normal comb development were selected from both male and female chickens, respectively to measure skeletal development, body size traits, and slaughter traits (Table S1). To evaluate the growth performance across groups with large and small comb areas, the comb length and comb height of the entire population were measured at 240 d for male and 300 d for female chickens, corresponding to market age. The comb area was determined by multiplying comb length by comb height. Thresholds for categorizing large and small comb groups were established using the maximum, minimum, and median comb areas within each population. Individuals with comb areas between (maximum - median)/2 + median and the maximum value were classified as the large comb group, while those with comb areas between the minimum value and (minimum + median)/2 were classified as the small comb group (Table 1). From the individuals within the threshold ranges, 12 chickens were randomly selected from each group to measure growth performance.

**Table 1:** Selection of threshold values for grouping large and small combs (mm^2^).

Term	Male chicken	Female chicken
max	10434.75	2026.93
median	8597.50	1116.77
min	2230.83	551.55
threshold for large comb group	9516.13	1571.85
threshold for small comb group	5414.16	834.16

At 240 days of age for male chickens and 300 days of age for female chickens, the comb length and comb height of the flock were measured, and the comb area was calculated as the product of these two measurements. The comb area was then used to group the chickens based on its extreme values. For the large comb group: individuals with comb areas between (maximum − median)/2+ median and the maximum value were selected. For the small comb group: individuals with comb areas between the minimum and (minimum + median)/2 were selected. From the population within these threshold ranges, 12 individuals were randomly selected from each of the large and small comb groups to measure growth performance.

### Histomorphological evaluation and meat quality measurement

The comb tissues were collected and fixed in 4 % paraformaldehyde for 24 h, dehydrated, sectioned into 5 μm slices, and embedded in paraffin. Hematoxylin and eosin (HE) staining was performed on the comb tissue sections for histological observation. The germinal layer, peripheral dermis, and keratin layer were observed. Tissue sections were examined and photographed under a microscope using a camera system. Breast and leg muscles were collected from the chickens to measure pH, meat color (L* value, a* value, and b* value), moisture, crude protein, crude fat, inosine monophosphate (IMP), muscle fiber diameter, and density.

### RNA extraction and qPCR with reverse transcription

Total RNA was isolated using an RNATrizol (Molecular Research Center, Cincinnati, OH) according to the manufacturer’s instructions. The quality of RNA was assessed using 1 % agarose gel electrophoresis, RNA concentration was analyzed using a Qubit® 2.0 Fluorometer (Life Technologies). A total of 1 μg of purified RNA was reverse transcribed using the PrimeScript™ RT Reagent Kit (Takara, Dalian, China). For qPCR with reverse transcription (RT–qPCR), an aliquot of the RT reaction was analysed with SYBR® Premix Ex Taq^TM^ (2 ×) and a Bio-Rad CFX96 real-time PCR system. Five biological replicates were set, with three technical replicates for each sample. Target transcript levels were normalized to β-actin. Relative expression was calculated using the 2^−(ΔΔCt)^ method, and primer information is listed in Table S2.

### Chicken serum GH and testosterone ELISA assay

Serum levels of growth hormone (GH) and testosterone were assayed by ELISA. GH was measured in serum collected from chickens with different comb sizes using the ELISA Kit (Jiangsu Baolai Biotechnology Co., Ltd. China). Testosterone was measured in serum using the ELISA Kit (Jiangsu Baolai Biotechnology Co., Ltd. China). The absorbance was measured at 450 nm using a microplate spectrophotometer, and analyzed using a standard curve.

### Statistical analysis

The results were presented as mean ± standard error of the mean (SEM). Statistical analysis was conducted using SPSS 27 software, while tissue section measurements and statistical evaluations were performed with ImageJ software. Data visualization was carried out using ggplot2 package in R. The Mantel test analysis was performed using the linkET package in R.

## Results

### Analysis of the comb, skeletal development, and carcass performance of Sichuan Mountaineous Black-bone chicken

**Fig. 1:**
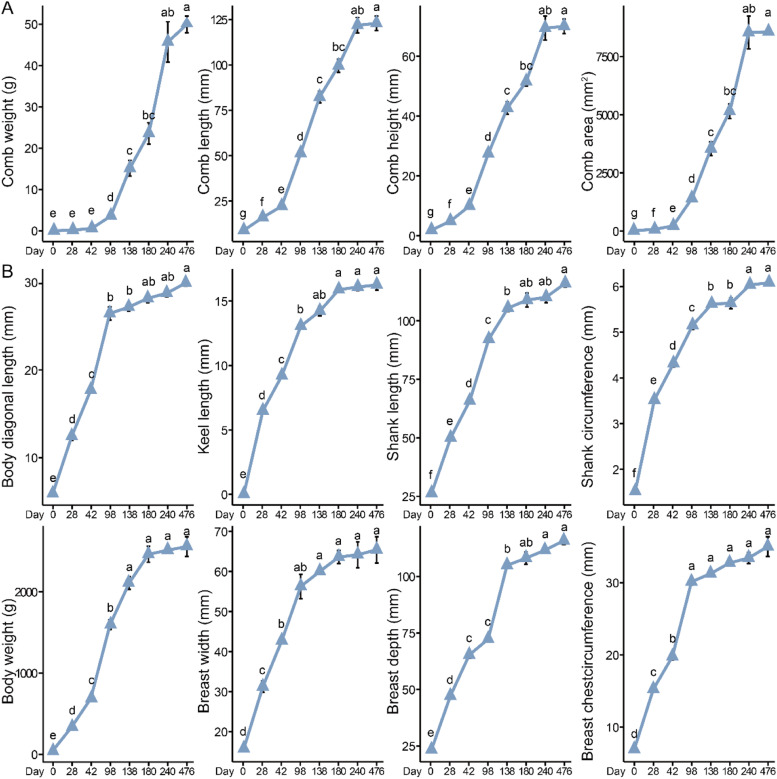
The changes in the comb and body measurements of male chickens from 0 to 476 days (n = 12). (A) Changes in comb weight, comb length, comb height, and comb area across different ages. (B) Changes in body diagonal length, keel length, shank length, shank circumference, body weight, breast width, breast depth, and breast circumference across different ages. Different time points were analyzed using one-way analysis of variance and Tukey or Dunnett multiple comparisons. Different letters indicate statistical differences at *P* < 0.05.

**Fig. 2:**
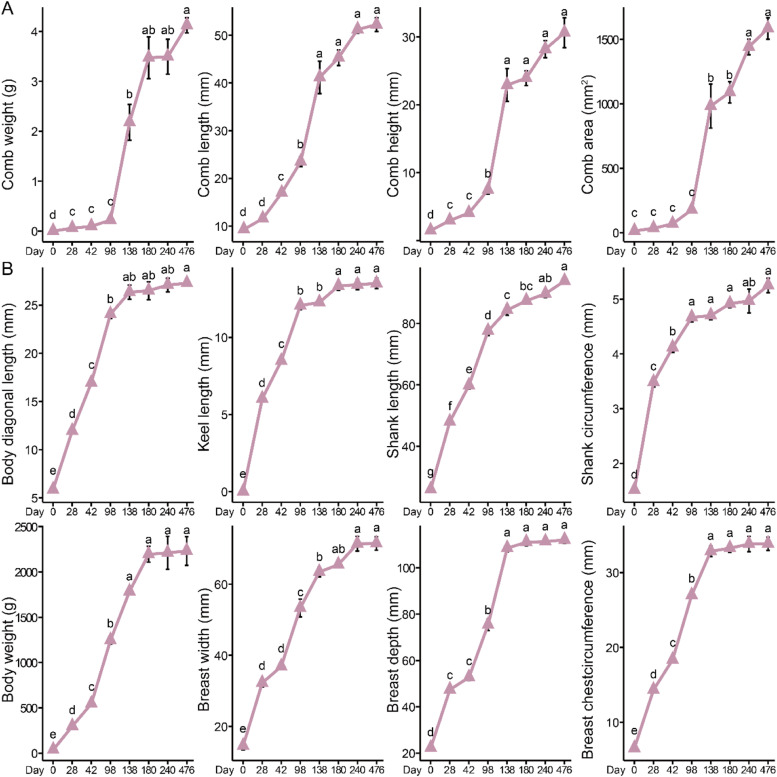
The changes in the comb and body measurements of female chickens from 0 to 476 days (n = 12). (A) Changes in comb weight, comb length, comb height, and comb area across different ages. (B) Changes in body diagonal length, keel length, shank length, shank circumference, body weight, breast width, breast depth, and breast circumference across different ages. Different time points were analyzed using one-way analysis of variance and Tukey or Dunnett multiple comparisons. Different letters indicate statistical differences at *P* < 0.05.

To investigate the comb, growth, and slaughter performance of Sichuan Mountaineous Black-bone chickens, we analyzed several key growth and development stages. In terms of the comb development pattern pattern followed an “S”-shaped curve (Fig. 1A and 2A). Before 42 days, comb development measured in terms of comb weight, comb length, comb height, and comb areaprogressed slowly.

As the chickens aged, comb growth gradually accelerated. In male chickens, comb growth began to decelerate after 180 days (Fig. 1A), whereas in female chickens, this slowdown occurred earlier, at 138 days (Fig. 2A). At 476 days of age, male chickens comb can weigh approximately 50 g, whereas female chickens comb weigh only about 4 g. In contrast, body size traits developed rapidly in the early stages but slowed during the middle and later stages. Specifically, for skeletal development in male chickens, body diagonal length, keel length, breast width, and breast circumference slowed after 98 days, these traits reached 26.43 mm, 13.05 mm, 52.26 mm, and 30.12 mm at 98 days of age. However, between 98 and 476 days of age, the comb length, height, area, and weight increased by only 3.48 mm, 3.20 mm, 9.17 mm, and 4.88 g, respectively, indicating a marked deceleration in growth rate during this period. While shank length, shank circumference, and breast depth, consistent with body weight, slowed at the next observed time point (138 days) (Fig. 1B). In female chickens, the growth of body diagonal length, keel length, and shank circumference began to slow at 98 days of age, reaching 24.05 mm, 12.07 mm, and 4.67 mm, respectively. Between 98 and 476 days, these traits increased by only 3.22 mm, 1.43 mm, and 0.58 mm, indicating a notable reduction in growth rate. Whereas shank length, breast width, breast depth, and breast circumference, consistent with body weight, showed a slower development trend after 138 days (Fig. 2B).

To examine the changes in slaughter traits of Sichuan Mountaineous Black-bone chickens at different key time points, we measured their performance at different time points. In male chickens, traits such as slaughter weight, breast muscle weight, leg muscle weight, testicular weight, half-eviscerated weight, and eviscerated weight followed an “S”-shaped growth pattern, with these indicators significantly increasing at 98 days or 138 days. However, other traits did not display distinct age-related trends. While breast muscle ratio and leg muscle ratio showed a slight upward trend with age, the changes were minimal (Figure S1). Similar patterns were observed in female chickens. However, the slaughter ratio in female chickens significantly decreased at 98 and 138 days, while other indicators were comparable to those in male chickens (Figure S2). Across all measures comb development, body size traits, and slaughter performance the growth curves for male and female chickens were largely similar. This suggests that Sichuan Mountaineous Black-bone chickens of both sexes follow a comparable developmental trajectory as they age.

### Developmental patterns of histomorphological structure of Sichuan Mountaineous Black-bone chicken comb

**Fig. 3:**
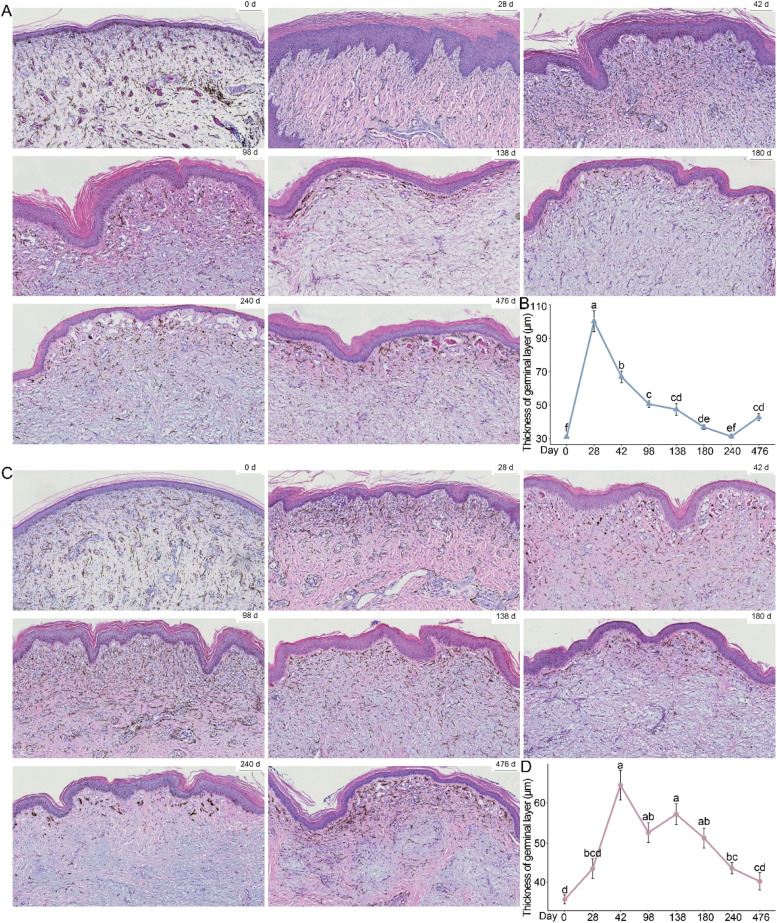
Histological morphology and germinal layer thickness statistics of the epidermis and dermis in the combs of Sichuan Mountaineous Black-bone chickens at different ages. (A) Representative HE-stained images of male chicken’s comb aged 0–476 days, scale bar = 100 nm. (B) Statistical analysis of germinal layer thickness in male chicken’s comb. One-way ANOVA followed by Tukey or Dunnett multiple comparisons was used for analysis at different time points. (C) Representative HE-stained images of female chicken’s comb aged 0–476 days, scale bar = 100 nm. (D) Statistical analysis of germinal layer thickness in female chicken’s comb. One-way ANOVA followed by Tukey or Dunnett multiple comparisons was used for analysis at different time points. Different letters indicate statistical differences at *P* < 0.05.

To investigate the developmental changes in the comb with age, we analyzed the comb tissue structure of Sichuan Mountaineous Black-bone chickens at several key developmental stages. The results showed that the comb tissue structure of both male and female chickens was identical. Due to the black comb characteristic of Sichuan Mountaineous Black-bone chickens, varying numbers of melanocytes were distributed within the comb tissue. As the comb developed, the number of sinusoidal capillaries in the peripheral dermis increased, their diameters gradually enlarged, and the number of red blood cells within them also increased. At 28 days, the germinal layer in both male and female chickens exhibited folds of varying sizes (Figs. 3A and 3C). Interestingly, the thickness of the germinal layer in male chickens peaked at 28 days and then gradually thinned with age (Fig. 3B). In contrast, the germinal layer thickness in female chickens increased with age, reaching its peak at 42 days, before gradually thinning (Fig. 3D). Notably, in the later stages of growth, the germinal layer thickness in both male and female chickens returned to approximately the same thickness as at hatch. In addition, we found that, starting from hatch, the germinal layer thickness in male chickens was significantly greater than that in female chickens at all observed time points, except at 42 days, 98 days, and 476 days (Figure S3).

### Histomorphological structure and hormone analysis of different sizes of chicken combs

**Fig. 4:**
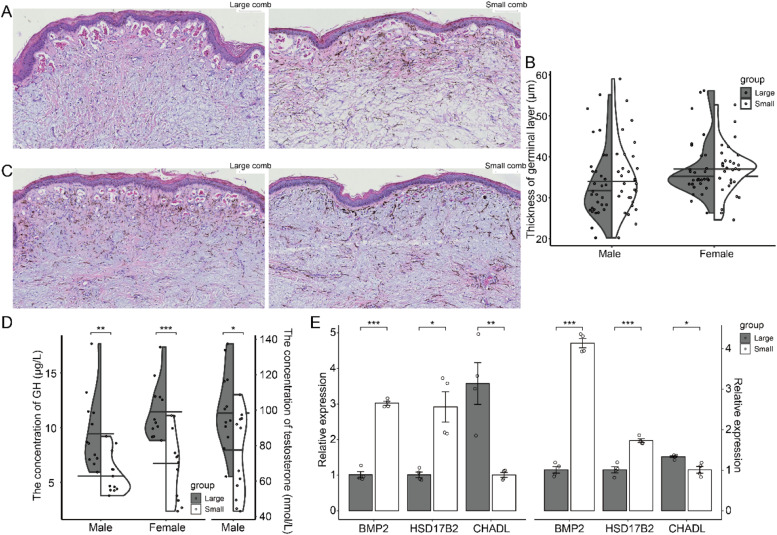
Tissue morphology and serum hormone analysis of Sichuan Mountaineous Black-bone chickens with different sizes of combs. (A) Representative HE-stained images of combs from male chickens with large and small combs. (B) Representative HE-stained images of combs from female chickens with large and small combs. (C) The germinal layer thickness is similar between male and female chickens with large and small combs (n = 12). (D) In male chickens, circulating GH levels are higher in the large comb group compared to the small comb group, while testosterone levels are similar; in female chickens, circulating GH levels are similar between different comb sizes. (E) Expression levels of comb development-related genes in the combs of male and female chickens with different comb sizes. Statistical significance was indicated as follows: *P < 0.05, ^⁎⁎^*P* < 0.01, ^⁎⁎⁎^*P* < 0.001; values without asterisks were not statistically significant. Comparisons between different comb sizes were performed using independent sample t-tests.

It was revealed in this study that the comb size varied within the same sex in Sichuan Mountaineous Black-bone chickens. Therefore, in this study, we categorized combs into large and small groups based on comb measurements at the market age. Subsequently, we analyzed the tissue structure of combs of different sizes. Regardless of sex, the combs in the large group had larger sinusoidal capillary diameters, a greater number of sinusoidal capillaries in the peripheral dermis, and more red blood cells within them compared to the small group. However, no differences in germinal layer thickness were observed between the large and small comb groups (Fig. 4A and 4C). In addition, we found that GH levels were elevated in the male chickens large-comb group, while no changes in testosterone levels were detected (Fig. 4D). In female chickens, no significant differences in GH levels were observed between the large and small comb groups (Fig. 4D). To further investigate the causes of comb size differences, we examined the expression of genes related to comb development. The results showed that *BMP2* and *HSD17B2* expression levels were significantly higher in the small-comb group compared to the large-comb group in both male and female chickens. Conversely, *CHADL* expression levels were significantly higher in the large-comb group (Fig. 4E).

### Comparison of comb, skeletal development, and slaughter performance of Sichuan Mountaineous Black-bone chickens with different comb sizes

To investigate whether there are differences in skeletal development and slaughter performance among chickens of different sizes, they were divided into two groups based on comb size for analysis. The results showed that, for male chickens, the skeletal development of the large-comb group was generally superior to that of the small-comb group, with significant increases in shank circumference, breast depth, and breast circumference. However, regarding slaughter traits, after normalization to slaughter weight, no significant differences were observed between the two groups. For female chickens, the skeletal development of the large-comb group also tended to be higher than that of the small-comb group, but the differences were not significant. Similarly, slaughter performance showed no significant differences, consistent with the results for the male chickens (Table 2).

**Table 2:** Analysis of comb, skeletal development, and slaughter performance of chickens with different comb sizes.

	Male chicken			Female chicken		
Term	Large	Small	*P*	Large	Small	*P*
**Comb traits**						
Comb length	**129.50±0.77**	**90.14±3.25**	⁎⁎⁎	**57.53±1.17**	**37.51±1.22**	⁎⁎⁎
Comb height	**76.12±0.65**	**48.34±2.03**	⁎⁎⁎	**30.67±0.56**	**18.90±0.63**	⁎⁎⁎
Comb area	**9855.38±81.47**	**4414.35±302.16**	⁎⁎⁎	**1764.74±49.97**	**705.34±25.34**	⁎⁎⁎
Comb weight	**49.40±2.25**	**19.91±1.76**	⁎⁎⁎	**3.53±0.14**	**1.63±0.11**	⁎⁎⁎
**Body size**						
Keel length	16.04 **±** 0.28	16.13 **±** 0.3	ns	**12.88±0.18**	**11.88±0.28**	⁎⁎
Body diagonal length	28.58 **±** 0.32	28.03 **±** 0.46	ns	26.23 **±** 0.56	25.63 **±** 0.33	ns
Shank length	110.52 **±** 2.49	105.86 **±** 1.26	ns	90.88 **±** 1.24	88.61 **±** 1.29	ns
Shank circumference	**6.00±0.08**	**5.69±0.07**	⁎⁎	5.01 **±** 0.06	4.89 **±** 0.08	ns
Breast width	63.77 **±** 1.23	61.76 **±** 2.10	ns	76.77 **±** 1.94	72.69 **±** 1.28	ns
Breast depth	**112.25±0.99**	**108.48±0.95**	*	101.53 **±** 1.64	100.75 **±** 1.59	ns
Breast circumference	**33.54±0.60**	**31.38±0.39**	⁎⁎	30.33 **±** 0.45	29.58 **±** 0.45	ns
**Body weight**						
Body weight	**2420.83±103.38**	**2071.67±75.12**	*	1983.00 **±** 67.12	1758.25 **±** 94.92	ns
**Slaughter performance**						
Slaughter weight	**2119.58±81.63**	**1790.83±61.13**	⁎⁎	1798.67 **±** 63.15	1624.33 **±** 86.32	ns
Breast muscle weight	230.43 **±** 13.74	197.57 **±** 11.14	ns	209.18 **±** 13.43	198.74 **±** 15.19	ns
Leg muscle weight	**434.33±21.18**	**347.06±14.99**	⁎⁎	258.75 **±** 11.02	235.32 **±** 13.85	ns
Half-eviscerated weight	**1954.58±79.26**	**1652.08±54.62**	⁎⁎	1512.17 **±** 50.79	1371.42 **±** 74.30	ns
Eviscerated weight	**1538.33±65.32**	**1305.00±45.39**	⁎⁎	1175.08 **±** 42.04	1071.75 **±** 64.11	ns
Testicular/ovarv weight	**34.48±2.14**	**23.05±2.49**	⁎⁎	41.83 **±** 1.49	37.75 **±** 3.07	ns
Slaughter ratio	87.76 **±** 0.82	86.58 **±** 0.98	ns	90.70 **±** 0.72	92.50 **±** 0.75	ns
Half-eviscerated weight ratio	80.85 **±** 0.84	79.89 **±** 0.85	ns	76.28 **±** 0.47	78.09 **±** 0.97	ns
Eviscerated weight ratio	63.58 **±** 0.75	63.04 **±** 0.43	ns	59.25 **±** 0.66	60.96 **±** 1.38	ns
Breast muscle ratio	14.96 **±** 0.56	15.06 **±** 0.63	ns	17.74 **±** 0.78	18.49 **±** 0.80	ns
Leg muscle ratio	28.23 **±** 0.67	26.59 **±** 0.61	ns	21.98 **±** 0.34	22.04 **±** 0.52	ns

The data are presented as mean ± SEM.

⁎ indicates *P* < 0.05,.

⁎⁎ indicates *P* < 0.01,.

⁎⁎⁎ indicates *P* < 0.001, and unmarked indicates no significance. Comparisons between different comb sizes were performed using independent sample t-tests.

In addition, meat quality was analyzed for both groups. For male chickens, significant differences in pH of the breast muscle were observed at 45 min and 24 h post-slaughter, with the large-comb group showing higher breast muscle moisture compared to the small-comb group. However, crude protein and crude fat levels were lower in the large-comb group. The IMP content and muscle fiber diameter of the large-comb group were higher than those of the small-comb group, but the differences were not significant. For leg muscle, apart from a significant difference in the a* value, other traits showed no significant differences. Although the large-comb group had higher crude protein, crude fat, and muscle fiber diameter in the leg muscle compared to the small-comb group, the differences were not significant. For female chickens, significant differences in breast muscle L* value were observed at 45 min and 24 h post-slaughter between the two comb size groups. The large-comb group had higher crude protein, IMP content (*P* < 0.05), and muscle fiber diameter compared to the small-comb group. For leg muscle, the a* value at 45 min and L* value at 24 h were significantly higher in the large-comb group. The large-comb group also showed lower moisture, and crude protein, crude fat content in the leg muscle compared to the small-comb group, although the differences were minor. Conversely, the IMP content and muscle fiber diameter were higher in the large-comb group (Table S3).

### Correlation analysis of circulating GH and testosterone with comb, skeletal development, and slaughter performance in Sichuan Mountaineous Black-bone chickens

**Fig. 5:**
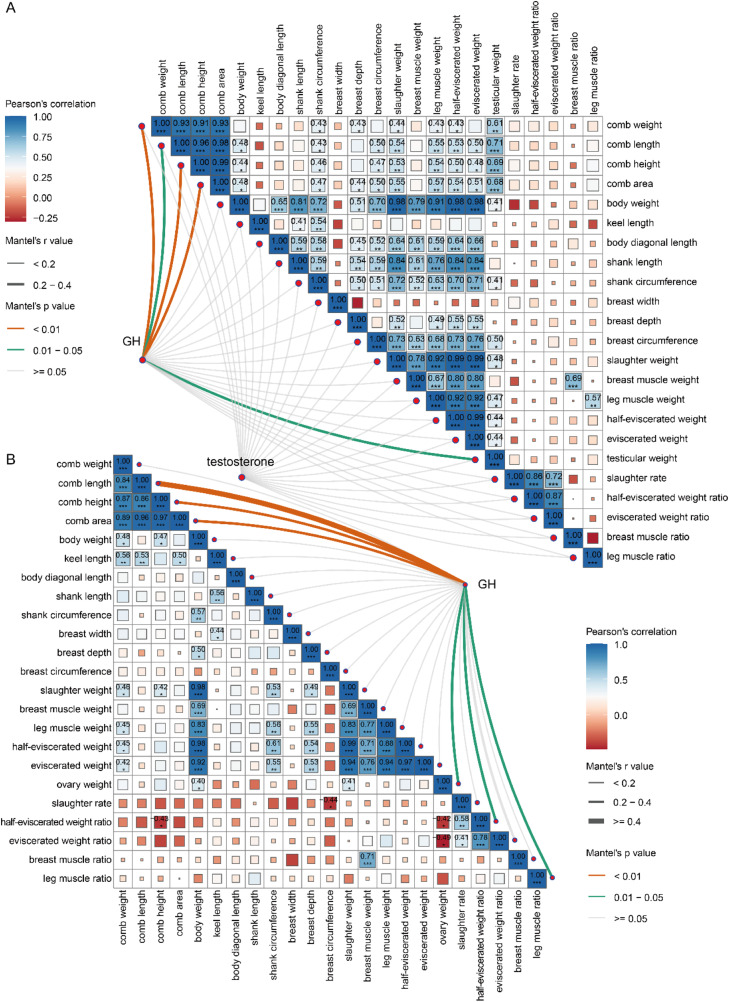
Mantel test of serum GH and testosterone with comb, body size development, and slaughter performance in Sichuan Mountaineous Black-bone chickens. (A) Mantel test analysis of circulating GH and testosterone with comb development, skeletal development, and slaughter traits in male chickens. (B) Mantel test analysis of circulating GH with comb development, skeletal development, and slaughter traits in female chickens. The Mantel test analysis was performed using the linkET package in R.

To further explore the relationship between hormones and comb development, skeletal growth, and slaughter performance, a Mantel test was conducted. In male chickens, growth hormone (GH) was significantly correlated with comb traits, including comb weight (r > 0.34, *P* < 0.01), length (r > 0.20, *P* < 0.05), height (r > 0.32, *P* < 0.01), and area (r > 0.28, *P* < 0.01), as well as with testicular weight (r > 0.23, *P* < 0.05). However, no strong correlation was found between GH and skeletal development (|r|<0.11, *P* > 0.05), nor between testosterone and comb (|r|<0.13) or skeletal development (|r|<0.15, *P* > 0.05) under the conditions of this study. Interestingly, body weight (r > 0.36), shank circumference (r > 0.43), breast circumference (r > 0.37), slaughter weight (r > 0.44), leg muscle weight (r > 0.43), half-eviscerated weight (r > 0.43), eviscerated weight (r > 0.40), and testicular weight (r > 0.61) were showed a positive correlation with comb development in male chickens (Fig. 5A). In female chickens, GH showed weak correlation with comb weight, skeletal development-related indicators, or slaughter traits (|r|<0.15, *P* > 0.05). However, GH has a significant impact on comb length (r > 0.40, *P* < 0.01), comb height (r > 0.20, *P* < 0.01), comb area (r > 0.27, *P* < 0.01), ovary weight (r > 0.31, *P* < 0.05), half-eviscerated weight ratio (r > 0.22, *P* < 0.05), and leg muscle ratio (r > 0.29, *P* < 0.05). Apart from this, we observed correlations between body weight (r = 0.48), keel length (r = 0.56), slaughter weight (r = 0.46), leg muscle weight (r = 0.45), half-eviscerated weight (r = 0.45), and eviscerated weight (r = 0.42) mainly with comb weight (Fig. 5B).

## Discussion

Understanding the mechanisms of morphogenesis and the functional significance of morphological traits remains a central goal of biological studies. In birds, the comb is a secondary sexual characteristic with critical roles in mate selection, health signaling, and reproductive performance (McGary et al., 2002; Johnsson et al., 2012; Boije et al., 2012). In the present study, we investigated the histomorphological development of the comb in Sichuan Mountaineous Black-bone chickens and its association with growth performance and hormone levels. By examining the comb development patterns at several key growth stages of Sichuan Mountaineous Black-bone chickens, we found that the combs of both male and female chickens develop slowly in the early stages, then accelerate in growth, and finally cease development around 476 days of age, overall, the development follows an “S-shaped” curve. The germinal layer, the innermost layer of the comb’s skin, consists mainly of basal cells and some keratinocytes. This layer is essential for maintaining the comb’s normal structure and function, supporting its growth and development, and contributing to overall comb health. Interestingly, no significant differences were observed in the thickness of the germinal layer among combs of varying sizes.

Since comb size is also influenced by potential genetic factors, we examined genes related to comb development. *BMP2* plays a well-established role in bone physiology and deposition (Salazar et al., 2016), while hyaluronic acid, the main component of the comb, is crucial in cartilage metabolism (Wu et al., 2018). In addition, cartilage serves as the precursor for all bone formation. We found that *BMP2* expression levels were downregulated in the comb tissue of the large-comb group of Sichuan Mountaineous Black-bone chickens, suggesting that it may inhibit comb growth by hindering cartilage development. The primary function of *HSD17B2* catalyzes the conversion of testosterone and dihydrotestosterone into their fewer active forms (Zhang et al., 2023). The expression level of *HSD17B2* in the large-comb group was significantly lower. Since comb development is regulated by sex hormones, *HSD17B2* may inactivate sex hormones, thereby negatively regulating comb development. *CHADL* regulates chondrocyte differentiation (Tillgren et al., 2015), and the expression level in the large-comb group was higher, suggesting that it may actively regulate comb growth by reducing cartilage metabolism.

Comb size has been linked to mating rank, skeletal development, and hormone levels (Verhulst et al., 1999; Cornwallis and Birkhead, 2007; Favati et al., 2021). In this study, male chickens with large combs exhibited elevated GH levels, which may have contributed to their greater body size (El-Attrouny et al., 2021). While testosterone is widely recognized as a key determinant of comb size, no differences in testosterone levels were detected between male chickens with large and small combs. This finding aligns with the hypothesis that testosterone may mediate a trade-off between ornamentation and immune capacity (Verhulst et al., 1999). In female chickens, somatic investment in the comb has been shown to reflect reproductive investment, with larger combs associated with increased skeletal development (Wright et al., 2008).

Our results revealed that male chickens with large combs demonstrated superior skeletal traits, including significantly greater shank circumference, breast depth, and breast girth. Similarly, female chickens with large combs displayed greater keel length, suggesting that comb size is a reliable indicator of overall growth in male chickens. However, no significant differences were observed in carcass yield between large- and small-comb groups, indicating that comb size may not directly influence carcass production. The relationship between comb size and skeletal traits appeared to be more pronounced in males.

To further explore the association between hormone levels, comb development, skeletal growth, and slaughter traits, the Mantel test was conducted. GH levels were found to be significantly correlated with comb development and testicular weight in male chickens, consistent with GH’s role in promoting growth and secondary sexual characteristics (Devesa et al., 1991; Strobl and Thomas, 1994). Interestingly, in this study, the testosterone levels did not show a significant correlation with comb development.

## Conclusion

In conclusion, the comb of Sichuan Mountaineous Black-bone chickens follows an “S”-shaped developmental pattern, with GH playing a central role in mediating comb growth and related skeletal traits, particularly in male chickens. In female chickens, comb weight positively correlated with body weight, keel length, and slaughter weight. These findings suggest that comb size can serve as a valuable phenotypic marker in breeding programs aimed at improving growth performance. Future studies will focus on elucidating the precise genetic and hormonal mechanisms regulating comb development to support targeted genetic selection in chickens.

## Ethics approval and consent to participate

The animal experimental procedures were approved by the Institutional Animal Care and Use Committee of Sichuan Agricultural University, China (2022. 12. 06. Certification No. SYXK2019-187), and all the experiments were conducted in accordance with the guidelines provided by the Sichuan Agricultural University Laboratory Animal Welfare and Ethics.

## Author's Role

Kunlong Qi, Juan He, and Felix Kwame Amevor: Conceptualization, formal analysis, methodology, and writing original draft; Kunlong Qi, Zheliang Liu and Chencan Zhai: Formal analysis, software, and methodology; Yingjie Wang, Liuting Wu and Gang Shu: Supervision; Felix Kwame Amevor, Gang Shu, Xiaoling Zhao: Conceptualization, data curation, supervision, and writing-review and editing. All authors have read and agreed to the published version of the manuscript.

## Disclosures

The authors declare that they have no known competing financial interests or personal relationships that could have appeared to influence the work reported in this paper.

## Appendix Supplementary materials

Image, application 1
