# Supplementary material for: Analysis of comb morphology in Sichuan Mountaineous Black-bone chickens and its correlation with growth performance
Source: Poult Sci. 2025 Apr 17;104(7):105168. doi: 10.1016/j.psj.2025.105168 (PMC12207823; doi:10.1016/j.psj.2025.105168)
Supplement: Supplementary file 1 [file mmc1.docx]

**Supplementary Figures and tables**


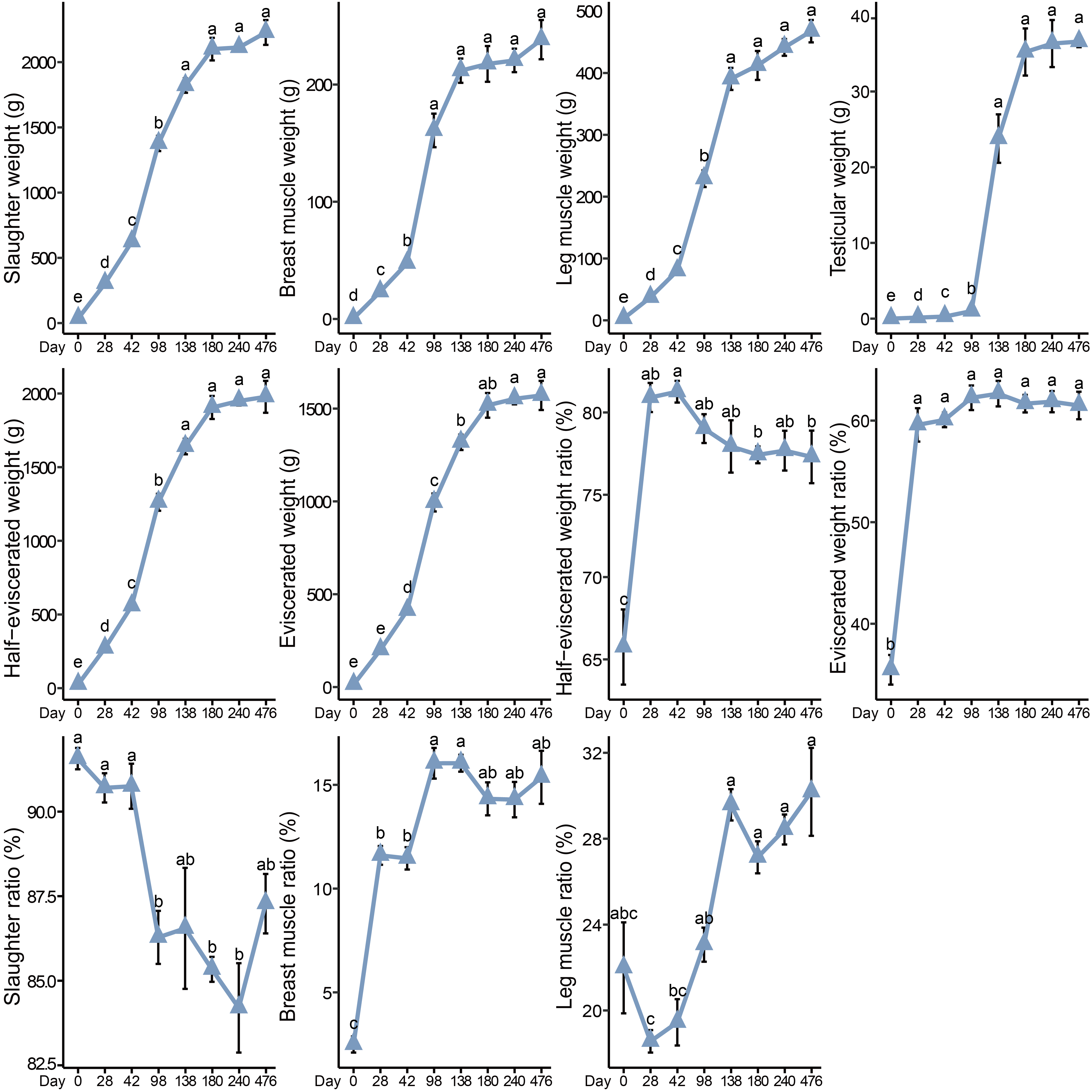


**Figure S1.** Changes in slaughter performance of male chickens (n = 12) from Day 0 to Day 476. Slaughter performance traits, including slaughter weight, breast muscle weight, leg muscle weight, testicular weight, half-eviscerated weight, eviscerated weight, half-eviscerated yield, eviscerated yield, slaughter yield, breast muscle yield, and leg muscle yield, were evaluated in male chickens across multiple time points from hatch (Day 0) to 476 days of age. A one-way analysis of variance (ANOVA) was performed to assess differences across time points, followed by Tukey’s or Dunnett’s post hoc test for multiple comparisons. Different superscript letters indicate statistically significant differences (*P* < 0.05).


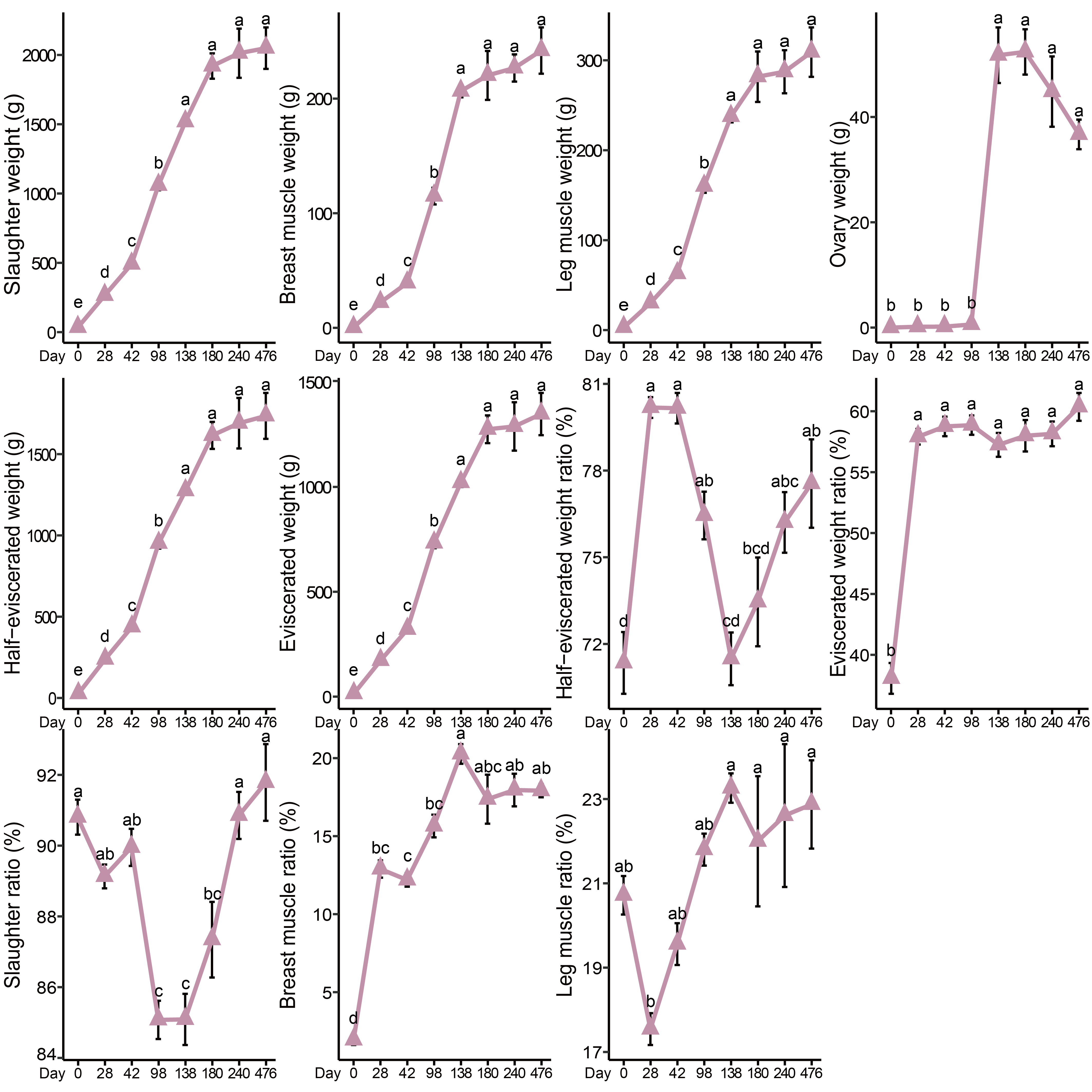


**Figure S2.** Changes in slaughter performance of female chickens (n = 12) from Day 0 to Day 476. Slaughter performance parameters, including slaughter weight, breast muscle weight, leg muscle weight, ovary weight, half-eviscerated weight, eviscerated weight, half-eviscerated yield, eviscerated yield, slaughter yield, breast muscle yield, and leg muscle yield, were assessed in female chickens from hatch (Day 0) to 476 days of age. Data were analyzed using one-way analysis of variance (ANOVA) across different time points, followed by Tukey’s or Dunnett’s multiple comparison tests. Different superscript letters denote statistically significant differences (*P* < 0.05).


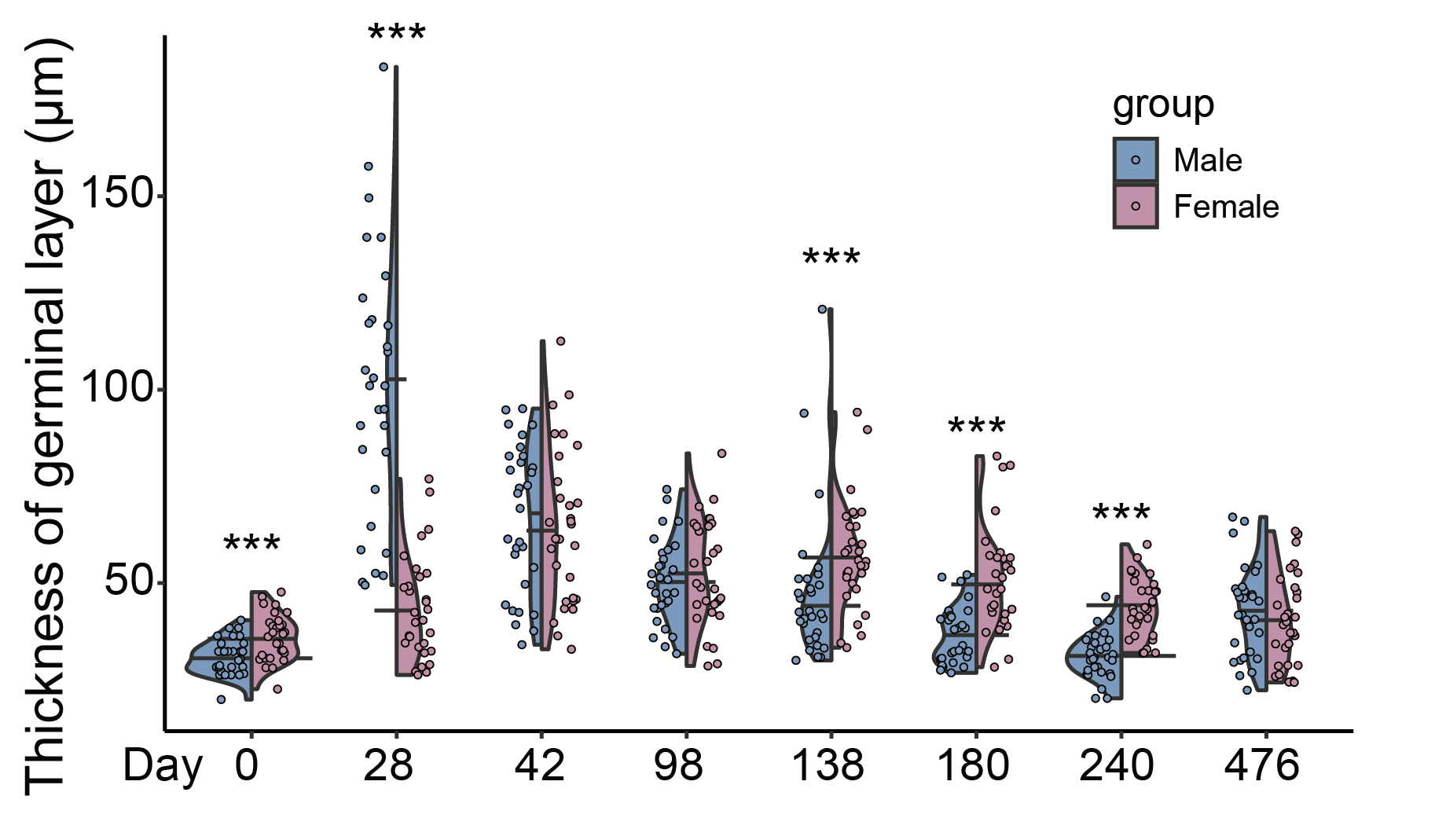


**Figure S3.** The thickness of the germinal layer was measured at various ages. Statistical significance was indicated as follows: ^*^*P* < 0.05, ^**^*P* < 0.01, ^***^*P* < 0.001; values without asterisks were not statistically significant. Comparisons between chickens with different comb sizes were conducted using independent sample t-tests.

**Table S1 Important development nodes of Sichuan Mountainous Black-bone chickens**

| **Days (d)** | **Developmental stage** |
| --- | --- |
| 0 | Chick’s hatch |
| 28 | Comb starts developing |
| 42 | Comb development accelerates |
| 98 | Growing period, the comb develops significantly |
| 138 | Reaching maturity stage |
| 180 | Comb development gradually slows down |
| 240 | Complete sexual maturity stage |
| 476 | Elimination of laying hens, cockscomb stop growing |

**Table S2 The primers information used in this study**

| **Gene name** | **Forward (5’-3’)** | **Reverse (5’-3’)** |
| --- | --- | --- |
| *BMP2* | CAGGATGAAGATAGCTGGT | CTTACGCTGTTTGTGTTTCG |
| *CHADL* | CCTTCACAAGCCTTCAAGAG | CTCCAGACTGTTTCAGCATT |
| *HSD17B2* | CTTAGGTGCTCGCAAACATT | TAACACAGCGGTGATTAAGGAT |
| *β-actin* | GAGAAATTGTGCGTGACATCA | CCTGAACCTCTCATTGCCA |

**Table S3 Comparison of meat quality in chicken combs of different sizes**

|  |  |  | **Male chicken** |  |  | **Female chicken** |  |
| --- | --- | --- | --- | --- | --- | --- | --- |
|  | **Term** | **Large** | **Small** | ***P*-value** | **Large** | **Small** | ***P*-value** |
|  | pH_45min_ | 5.84±0.04 | 5.69±0.03 | ** | 5.91±0.04 | 5.81±0.05 | ns |
|  | L*_45min_ | 36.11±0.91 | 37.72±1.24 | ns | 44.3±0.81 | 40.51±1.04 | ** |
|  | a*_45min_ | 7.77±0.72 | 6.04±0.46 | ns | 1.85±0.14 | 1.66±0.22 | ns |
|  | b*_45min_ | 3.32±0.45 | 3.23±0.14 | ns | 5.68±0.4 | 4.81±0.35 | ns |
|  | pH_24h_ | 5.81±0.02 | 5.63±0.03 | *** | 5.62±0.04 | 5.78±0.13 | ns |
|  | L*_24h_ | 36.16±1.07 | 38.13±0.88 | ns | 47.36±1.7 | 41.37±0.87 | ** |
| Breast muscle | a*_24h_ | 8.71±0.78 | 7.04±0.56 | ns | 2.17±0.34 | 1.76±0.21 | ns |
|  | b*_24h_ | 3.43±0.44 | 3.42±0.36 | ns | 4.72±0.19 | 4.65±0.35 | ns |
|  | Moisture | 67.98±0.51 | 65.8±0.75 | * | 66.96±0.42 | 66.94±0.41 | ns |
|  | Crude protein | 26.25±0.32 | 27.21±0.25 | * | 25.09±0.28 | 24.96±0.33 | ns |
|  | Crude fat | 4.73±0.03 | 5.01±0.08 | ** | 5.02±0.08 | 5.05±0.06 | ns |
|  | IMP | 2.02±0.16 | 1.92±0.08 | ns | 2.7±0.35 | 1.63±0.17 | * |
|  | Fiber diameter | 30.85±2.94 | 28.5±0.71 | ns | 43.31±2.62 | 40.38±1.65 | ns |
|  | pH_45min_ | 5.98±0.04 | 5.87±0.04 | ns | 6.03±0.03 | 6.01±0.07 | ns |
|  | L*_45min_ | 28.24±0.67 | 29.52±0.59 | ns | 32.99±0.92 | 31.37±0.78 | ns |
|  | a*_45min_ | 12.09±0.66 | 11.02±0.75 | ns | 9.38±0.32 | 6.57±0.55 | *** |
|  | b*_45min_ | 3.07±0.21 | 2.95±0.14 | ns | 5.22±0.33 | 4.56±0.29 | ns |
|  | pH_24h_ | 5.84±0.03 | 5.77±0.03 | ns | 5.85±0.06 | 5.92±0.03 | ns |
|  | L*_24h_ | 30.1±0.52 | 30.29±0.66 | ns | 40.78±1.7 | 35.98±0.97 | * |
| Leg muscle | a*_24h_ | 12.94±0.61 | 11.06±0.44 | * | 3.96±0.38 | 4.39±0.47 | ns |
|  | b*_24h_ | 3.4±0.23 | 3.38±0.28 | ns | 3.87±0.36 | 3.54±0.4 | ns |
|  | Moisture | 65.95±0.52 | 66.68±0.52 | ns | 65.17±0.44 | 66.34±0.66 | ns |
|  | Crude protein | 24.93±0.39 | 24.85±0.28 | ns | 22.32±0.48 | 23.08±0.46 | ns |
|  | Crude fat | 5.08±0.08 | 4.97±0.08 | ns | 5.07±0.06 | 5.2±0.06 | ns |
|  | IMP | 2.96±0.25 | 3.24±0.4 | ns | 3.01±0.42 | 2.3±0.19 | ns |
|  | Fiber diameter | 32.07±1.29 | 31.31±1.08 | ns | 36.53±0.99 | 35.13±2.18 | ns |

IMP: inosine monophosphate. Data are expressed as mean ± SEM. Statistical significance is denoted as follows: ^*^*P* < 0.05, ^**^*P* < 0.01, ^***^*P* < 0.001, and ns indicates no significant difference. Comparisons between chickens with different comb sizes were conducted using independent sample t-tests.
